# Staging of Alzheimer's Pathology in Triple Transgenic Mice: A Light and Electron Microscopic Analysis

**DOI:** 10.4061/2010/780102

**Published:** 2010-07-15

**Authors:** Kwang-Jin Oh, Sylvia E. Perez, Sarita Lagalwar, Laurel Vana, Lester Binder, Elliott J. Mufson

**Affiliations:** ^1^Department of Neurological Sciences, Rush University Medical Center, Chicago, IL 60612, USA; ^2^Department of Cell and Molecular Biology, Feinberg School of Medicine, Northwestern University, Chicago, IL 60611, USA

## Abstract

The age-related pathological cascade underlying intraneuronal tau formation in 3xTg-AD mice, which harbor the human APP_Swe_, PS1_M126V_ , and Tau_P301L_ gene mutations, remains unclear. At 3 weeks of age, AT180, Alz50, MC1, AT8, and PHF-1 intraneuronal immunoreactivity appeared in the amygdala and hippocampus and at later ages in the cortex of 3xTg-AD mice. AT8 and PHF-1 staining was fixation dependent in young mutant mice. 6E10 staining was seen at all ages. Fluorescent immunomicroscopy revealed CA1 neurons dual stained for 6E10 and Alz50 and single Alz50 immunoreactive neurons in the subiculum at 3 weeks and continuing to 20 months. Although electron microscopy confirmed intraneuronal cytoplasmic Alz50, AT8, and 6E10 reaction product in younger 3xTg-AD mice, straight filaments appeared at 23 months of age in female mice. The present data suggest that other age-related biochemical mechanisms in addition to early intraneuronal accumulation of 6E10 and tau underlie the formation of tau filaments in 3xTg-AD mice.

## 1. Introduction

During the last several years numerous transgenic animal models of Alzheimer's disease (AD) have been engineered to examine the effects of the two major AD neuropathological hallmarks, amyloid plaques, and neurofibrillary tangles (NFT) on neurodegeneration. The vast majority of these AD transgenic mice overexpress a mutant human amyloid-beta (A*β*) precursor protein (APP) gene alone or in combination with a mutated presenilin (PS) gene resulting in the presence of brain extracellular amyloid plaques, which are mainly formed by the accumulation of insoluble A*β* species [1–6]. Since overexpression of amyloid-beta peptide did not recapitulate all of the neuropathological features of AD, additional models were created adding mutant tau transgenes. For example, to further evaluate the pathogenic mechanisms underlying NFT formation, transgenic mouse models have been generated to harbor a mutant human tau gene found in frontotemporal dementia or Pick's disease (P301L or P301S). These mutant mouse models display NFT-like structures consisting of abnormal cytoskeletal tau protein aggregates in the central and peripheral nervous systems [4, 7–10]. Recently, a triple transgenic mouse (3xTg-AD) harboring the human APP_Swe_, PS1_M146V_, and Tau_P301L_ gene mutations was developed, displaying accumulation of both intracellular A*β*  and tau in an age-dependent manner within the cortex, hippocampus, and amygdala [11–14], and to a lesser degree, in the brainstem [15]. 

Immunohistochemical studies using immersion-fixed 3xTg-AD mouse tissue have shown that intracellular A*β*  precedes the appearance of tau pathology, developing A*β* deposits at 6 months and intraneuronal tau pathology at 9 months of age [11, 12, 14]. Recent reports have demonstrated that the development of A*β* plaques differed between 3xTg-AD mouse colonies as well as between male and female mice [16, 17]. Furthermore, rostral-caudal differences in the onset of tau pathology have been reported, but only in male 3xTg-AD mice [17]. Virtually no ultrastructural analysis of AD pathology in these mice has been described. 

Therefore, in the present study we performed a systematic detailed evaluation of the evolution of tau conformation and phosphorylation events beginning at 3 weeks of age using perfusion-fixed tissue at the light and electron microscopic level to more completely define the cascade of amyloid and tau pathology in male and female 3xTg-AD mice. The data derived from this study provide novel information underlying the temporal progression of amyloid and tau pathology within the cortex, hippocampal/subicular complex, and the amygdala that is pivotal in determining the selective vulnerability of neurons during the life span of male and female 3xTg-AD mice. This data is critical for the design of future experiments to address pharmacological, mechanistic, behavioral, and gender questions in studies using this widely used mouse model of AD.

## 2. Materials and Methods

### 2.1. Transgenic Mice

A colony of homozygous 3xTg-AD and nontransgenic (ntg) mice were generated from breeding pairs provided by Dr. Frank LaFerla, University of California Irvine. These transgenic mice harboring the human APP_Swe_, PS1_M146V_, and Tau_P301L_ mutations exhibit intraneuronal and extracellular amyloid pathology as well as tau pathology [12]. At least 4 male and 4 female juvenile (3 weeks), young (2-3 months), adult (4–6 months), middle-aged (8-9 months), and old (18–20 months) 3xTg-AD and *non-transgenic (ntg)* mice were examined. In addition young, middle-aged and old female 3xTg-AD mice were used for electron microscopic examination. Animal care and procedures were conducted according to the National Institutes of Health Guide for Care and Use of Laboratory Animals.

### 2.2. Fixation Protocol

Mice were anesthetized with an injection of ketamine/xylazine (100 mg/kg/5.0 mg/kg) and transcardially perfused for 2 minutes with 0.9% physiological saline followed by a solution containing 4% paraformaldehyde and 0.1% glutaraldehyde in 0.1 M phosphate buffer (PB) for 5 minutes (~50 ml) and then post-fixed in the same solution for 24 hours at 4°C. Since many transgenic mice studies use immersion-fixed brain tissue and considering that tau antigenicity is time and fixation sensitive [18–26], another group of mice was transcardially perfused with physiological saline and their brains hemisected and immersion-fixed for 24 hours in the same fixation solution. All brains were cryoprotected in 30% sucrose, sectioned on a sliding microtome at 40 micron thickness, and stored in a solution consisting of 30% glycerin, 30% ethylene glycol, in 0.1 M phosphate buffer at −20°C until processed for immunohistochemistry.

### 2.3. Immunohistochemistry

Tissues from immersion or perfusion fixed brains were processed as free-floating sections and immunostained for anti-hAPP/A*β* reactive amino acid residue 1–17 of beta-amyloid (6E10; 1 : 2,000 dilution, Covance, N.J.), the tau conformational antibodies Alz50 (66kD), MC1 (66kD) (1 : 10,000, 1 : 250 dilution, resp., both gifts from Dr. Peter Davies, Albert Einstein School of Medicine, N.Y.), and the phosphoepitope tau antibodies AT180 (~66 kD), AT8 (~66 kD) (both at 1 : 1000 dilution, Thermofisher, Waltham, MA) and PHF-1(57–67 kD) (1:10000 dilution; gift from Dr. Peter Davies, Albert Einstein College of Medicine) at each age examined. The specificity of each of the antibodies used in this study was previously characterized by western blot by others (6E10 (Covance), AT180 [27, 28], AT8 [29], PHF-1 [30–32], Alz50 [33–36], and MC1 [34, 35]). Prior to staining, sections were washed 3 × 10 min in phosphate buffer and 3 × 10 min in Tris-buffered saline (TBS) to remove cryoprotectant before a 20-minute incubation in 0.1 M sodium metaperiodate (Sigma, St. Louis, MO) in TBS to quench endogenous peroxidase activity. Tissue was then permeablized 3 × 10 minutes in TBS containing 0.25% Triton-X (Thermofisher, Waltham, MA) and blocked in the same solution containing 3% goat serum for 1 hour. Sections were incubated with appropriate antibody dilutions overnight on an orbital shaker at 45 RPM at room temperature in 0.25% Triton X-100, 1% goat serum solution. The next day, tissue was washed 3 × 10 min in TBS containing 1% goat serum prior to incubation with appropriate secondary antibody (see Table 1) at a 1 : 200 dilution for 1 hr. Following 3 × 10 minutes washes in TBS, sections were incubated in Vectastain ABC kit (Vector Labs, Burlingame, CA) in TBS for 1 hour. Tissue was then rinsed 3 × 10 minutes in 0.2 M sodium acetate, 1.0 M imidazol buffer, pH 7.4, and developed in acetate-imidazol buffer containing 0.05% 3,3'-diaminobenzidine tetrahydrochloride (DAB, Sigma, St Louis, MO). For comparison across ages sections from animals of different ages were immunostained at the same time and with the same duration of DAB reaction. The reaction was terminated in acetate-imidazol buffer, tissue mounted on glass slides, dehydrated through graded alcohols (70–95–100%, 3 × 5 min), cleared in xylenes (3 × 5 min), and coverslipped with DPX (Biochemica Fluka, Switzerland). Cytochemical control sections consisted of (1) tissue from ntg mice which were processed in a manner identical to the immunohistochemical procedures described above for 3xTg-AD animals, (2) tissue from 3xTg-AD as previously described with the exception of the primary antibody, and (3) a preadsorption control consisting of a 100 fold amount of A*β*_1-42_ (US Peptides, Rancho Cucamonga, CA) incubated with 6E10 in TBS containing 0.25% Triton X-100, 1% goat serum overnight at room temperature. The preadsorbed serum was used in place of the 6E10 antibody in the immunohistochemistry protocol. Since the antigen used to create the tau monoclonal antibodies obtained from commercial sources was unavailable, only the 6E10 preadsorbtion control was performed. Brightfield images were acquired using a Nikon Optiphot microscope.

### 2.4. 6E10 and Alz50 Dual Immunofluorescent Staining

Selected sections were fluorescently double-labeled for 6E10 and Alz50 using the above protocol with the following modifications [15]. All steps prior to incubation of primary antibody were as described above with the exclusion of the quenching of endogenous peroxidase activity with 0.1 M sodium metaperiodate. Tissue was incubated with primary antibody overnight (6E10; 1 : 200 dilution). After rinsing 3 × 10 minutes in TBS, tissue was incubated with Cy3-conjugated goat antimouse IgG Fc*γ* Subclass 1 specific secondary antibody (Jackson Immunoresearch; West Grove, PA) for 2 hours in the dark. Sections were washed 3 × 10 minutes in TBS and then incubated with Alz50 (1 : 1000) overnight in the dark. Tissue was again rinsed 3 × 10 minutes and then incubated in Cy2-conjugated goat antimouse IgM *μ*-chain specific secondary antibody (1 : 200 dilution) for 2 hours in the dark. Immunofluorescence was visualized using a Zeiss Axioplan 2 microscope using excitation filters at wavelengths 489 and 555 nm and emission filters at 505 and 570 nm for Cy2 and Cy3, respectively. Florescent images were stored on a computer and brightness and contrast was enhanced using GIMP Version 2.6.7. Double immunofluorescence staining for 6E10 and phosphotau markers were not successfully achieved using methods to block the cross reactivity between monoclonal IgG1 subclass primary antibodies (see Table 1).

### 2.5. Immunoelectron Microscopy

To determine ultrastructural localization of intraneuronal A*β* and tau immunoreactivity, we performed an immunoelectron microscopic analysis of hippocampal/subicular neurons obtained from female homozygous at 2- (*n* = 2), 9- (*n* = 2), and 23- (*n* = 2) month-old 3xTg-AD mice. Each animal was transcardially perfused with 4% paraformaldehyde/0.1% glutaraldehyde in phosphate buffer, brains were removed from the skull and immersion post-fixed in the same fixative overnight at 4°C and cut into 80 micron thick horizontal sections using a Vibratome-1500 (Vibratome, Saint-Louis, MO). Tissue containing the hippocampal/subicular complex was immunostained using the antibodies directed against A*β*/APP (6E10, 1 : 2000 dilution), the conformation specific tau antibody Alz50 (1 : 10,000 dilution), and the phosphospecific (Ser202/Thr205) antibody AT8 (1 : 1000 dilution). The chromogen DAB was used to visualize each antibody.

### 2.6. Transmission Electron Microscopy (TEM)

Immunostained sections were post-fixed in 1% OsO_4_ for 45–60 minutes, dehydrated and embedded in Epoxy resin. Immunoreactive profiles within the hippocampal/subicular complex were selected using light microscopy, ultrathin sections (50–70 nm) were cut using a Leica Ultracut UCT (Leica Microsystems Inc, Bannockburn, IL) microtome, and the majority of sections counter-stained with 2% uranyl acetate and lead citrate. TEM sections were viewed and photographed with a JEOL transmission electron microscope. Immunostaining appeared as an electron-dense precipitate (dark-black) in neurons and other profiles within hippocampal-subicular complex of mutant mice. Cortical brain tissue obtained at autopsy from an 82-year-old male AD patient was immersion fixed in 4% paraformaldehyde and processed for TEM without antibody staining to compare the ultrastructure of human AD pathology to that found in 3xTg-AD mice.

## 3. Results

### 3.1. General Considerations

**Figure 1:**
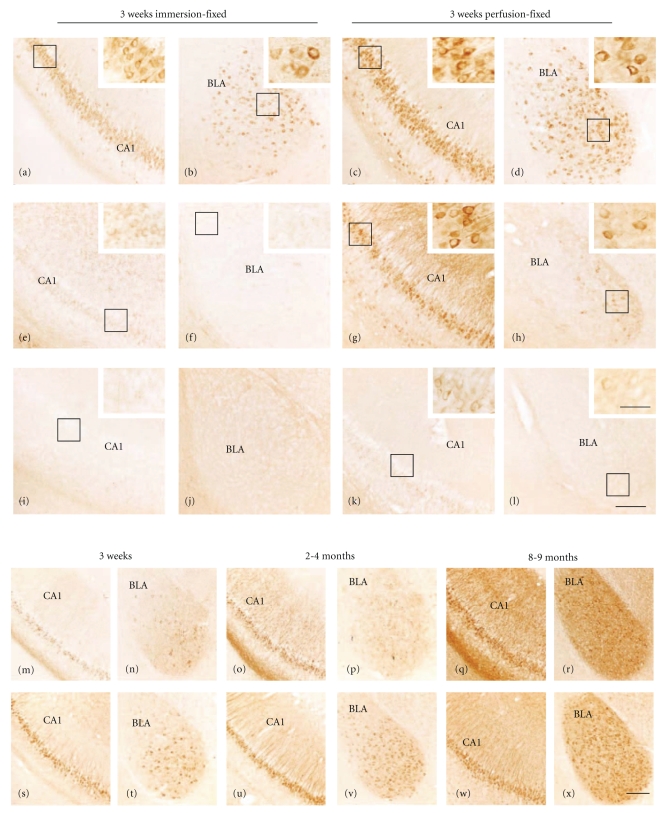
Photomicrographs of 6E10, AT8, PHF-1, Alz50, and AT180 intraneuronal and neuropil immunostaining in the CA1 field of the hippocampus and basolateral nucleus of the amygdala (BLA) at 3 weeks, 2–4-, and 8-9-month-old male 3xTg-AD mice. Note the differences in immunoreactivity between immersion and perfusion-fixed tissue in 3-week-old 3xTg-AD mice for 6E10 ((a)–(d)), AT8 ((e)–(h)), and PHF-1 ((i)–(l)). Alz50 ((m)–(r)) and AT180 ((s)–(x)) immunoreactivity was increased with age-dependent increases in the ventral hippocampus, ((m), (o), (q), and (s), (u), (w)) and basolateral amygdala, ((n), (p), (r) and (t), (v), (x)). Abbreviation: DG: dentate gyrus. Scale bar = 100 *μ*m.

In the present study forebrain sections from male and female 3-week, 2–4, 5-6, 8-9, and 18–20-month-old 3xTg-AD and age-matched ntg mice were immunostained using well-characterized antibodies directed against 6E10, tau conformational epitopes (Alz50 and MC1), and phosphotau epitopes AT180 (phosphothreonine 231), AT8 (phosphoserine 202/205), and PHF-1 (phosphoserine 396/404). In general, an age-related increase in intraneuronal and neuropil immunoreactivity in the hippocampal-subicular complex and amygdala were seen for all antibodies examined (Figure 1). The exception was MC1, where immunoreactivity decreased after 2–4 months of age (see Figure 6). Our findings revealed that transcardial but not immersion fixation dramatically enhanced the immunovisualization of AT8 (Figures 1(e)–1(h)) and PHF-1 (Figures 1(f)–1(l)) as early as 3 weeks of age. By contrast, AT180 and Alz50 immunoreactivity was not affected by the fixation procedure. Therefore all morphological analyses and photomicrographs subsequent to Figure 1 were derived from transcardially fixed tissue. The colocalization of 6E10 and tau antibodies using fluorescence was achieved successfully for 6E10 (IgG1) and Alz50 (IgM), but not for 6E10 and AT180 (IgG1) or AT8 (IgG1) due to the cross reactivity of their primary antibodies. Therefore, the comparative analyses of the distribution of 6E10 and phosphotau markers were performed using DAB-reacted tissue sections at the light microscopic level. All immunohistochemical controls failed to display cellular or plaque reactivity beyond background levels for any tau antigen or 6E10 independent of fixation, age, and gender.

### 3.2. Staging 6E10 Immunoreactivity

**Figure 2:**
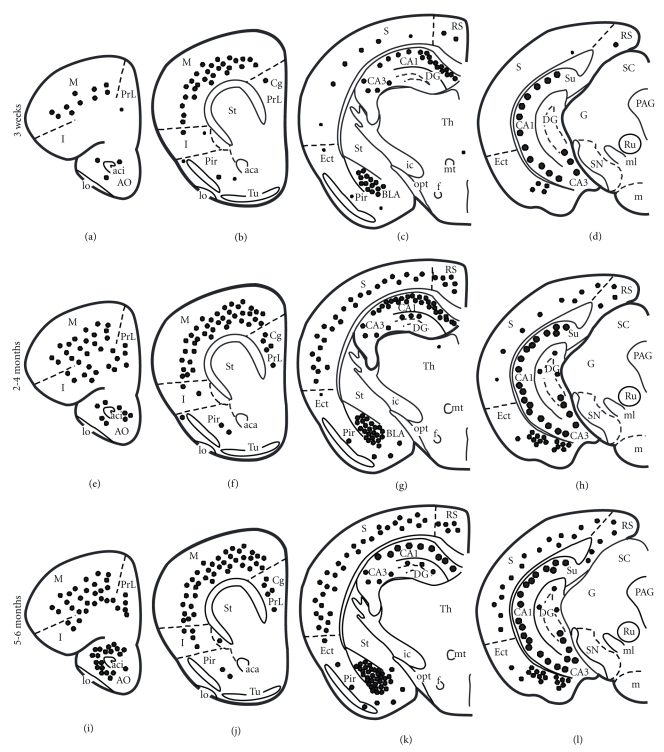
Schematic drawings representing the distribution of 6E10-ir neurons in 3-week, 2-, and 5-month-old perfusion-fixed 3xTg-AD transgenic mice. Note the age-related increase in the number of 6E10 positive neurons in the cortex, hippocampus, and amygdala. Abbreviations: Aca: anterior commissure, anterior; aci: anterior commissure, intrabulbar, A: amygdala; AO: anterior olfactory nucleus; BLA: basolateral nucleus of the amygdala; CA1: CA1 hippocampal subfield; CA3: CA3 hippocampal subfield; Cg: cingulate cortex; DG: dentate gyrus; Ect: ectorhinal cortex; F: fornix; G: geniculate nucleus; lo: lateral olfactory tract; I: insular cortex, ic: internal capsule; m: motor cortex; m: mammillary complex; ml: medial lemniscus; mt: mammillothalamic tract; opt: optic tract; PAG: periaqueductal gray, Pir: piriform cortex; Pr:L prelimbic cortex; Rn: red nucleus; RS: retrosplenial cortex; S: sensory cortex; SC: superior colliculus; SN: substantia nigra; St: striatum; Su: subiculum; th: thalamus; tu: olfactory tubercle. Small dots = 1–5 positive neurons, medium-size dots = 10–20 positive neurons, and large dots = 100 positive neurons.

**Figure 3:**
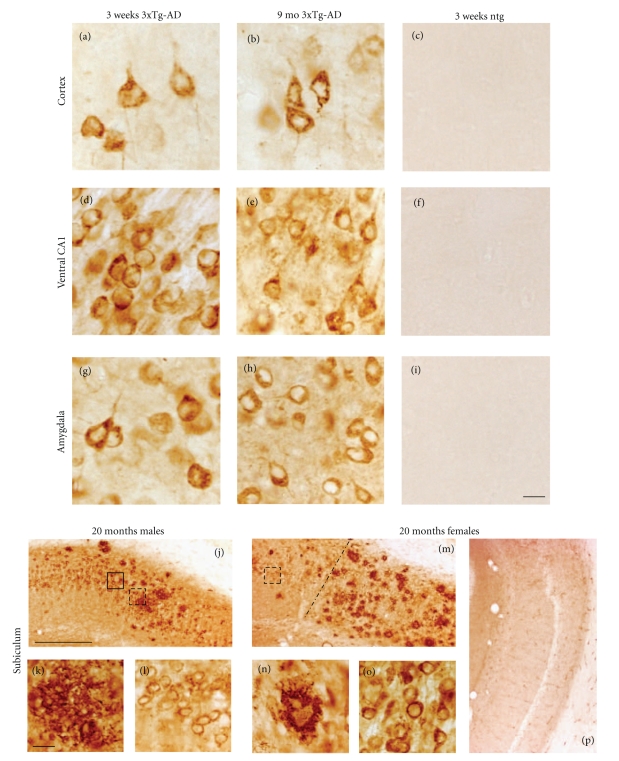
Photomicrographs showing 6E10 intraneuronal immnostaining in the cortex ((a)–(c)), ventral CA1 ((d)–(f)), BLA ((g)–(i)), and subiculum ((j)–(o)) in 3-week, 9- and 20-month-old 3xTg-AD mice. Round-or oval-shaped 6E10-ir neurons were found at the youngest age in all brain regions examined independent of fixation ((a), (d), (g)). At 9 months some 6E10 stained neurons appeared shrunken with a hollow center ((b), (e), (h)). 6E10 immunopositive plaques were found throughout the subiculum of female (j), and to a lesser extent in male mutant mice (m) at 20 months of age. Solid and dashed boxed areas in panels (j) and (m) outline a 6E10-ir plaque (k) and CA1-ir neurons shown at higher magnification in (l) and (o), respectively. Dashed line in panel m demarks the CA1 field from the subiculum. Higher-power photomicorgraph shows a posterior subicular 6E10 positive plaque in a female 20-month-old mutant mouse (n). Tissue processed following 6E10 antibody peptide preadsorbtion shows a lack of intra-or extraneuronal staining in CA1 or subiculum (P). Note the more diffuse compared to the compact nature of plaques found in male-versus female-aged mutant mice. Ntg mice failed to display 6E10 immunoreactivity (c, f, i) at 3 weeks of age. Scale bar in ((a)–(i)); ((k), ((l), (n), (o)) = 20 *μ*m, ((j), (m)) = 100 *μ*m.

The 6E10 antibody, which recognizes amino acids 3–8 of the A*β* sequence, revealed the greatest number and topographic distribution of immunoreactive (-ir) neurons in 3xTg-AD mice compared to the other antibodies we examined. There was an age-related increase in the extent of 6E10-ir neurons in all regions examined (Figure 2). Beginning at 3 weeks of age, intraneuronal 6E10-ir neurons were found in lamina 3 and 5 of the fronto-parietal cortex, deep layers of the cingulate cortex, CA fields of the hippocampus, subiculum, and the basolateral amygdala (BLA) in 3xTg-AD mice at each age independent of gender (Figure 2). 6E10-ir neurons, particularly in the hippocampal CA1 subfield, appeared round or oval in shape with many cells showing a thin ring of immunoreactivity located within the somatodendritic compartment (Figures 3(a), 3(d), and 3(g)). However, a number of cells in the cortex and amygdala had thickened perinuclear staining (Figures 3(d) and 3(a)), which became more prominent by 9 months of age (Figure 3(b) and 3(h)). Beginning at 9 months of age there was neuronal shrinkage of 6E10-ir neurons in 3xTg-AD mice (Figures 3(b), 3(e), and 3(h)), which continued at least until 18–20 months of age where many neurons displayed blunted dendrites, as well. 

Although no dramatic gender differences in intraneuronal 6E10 staining were seen prior to 8-9 months, A*β* plaque deposits were visualized for the first time in the subiculum of 8-9-month-old female, but not male mutant mice (data not shown). By 18–20 months both genders displayed A*β* plaque deposits in the subiculum (Figures 3(j), 3(k), 3(m), and 3(n)) and hippocampus (data not shown). A*β* plaques appeared smaller and more diffuse in male than seen in the female subiculum of 3xTg-AD mice (Figures 3(j) and 3(k)). In fact, female CA1 neurons lost their dendritic 6E10-ir extensions into the substantia radiatum (Figure 3(o)) while similar processes remained present in male mutant mice (Figures 3(l) and 3(o)).

### 3.3. Staging of Tau Pathology

#### 3.3.1. Alz50 Immunoreactivity

**Figure 4:**
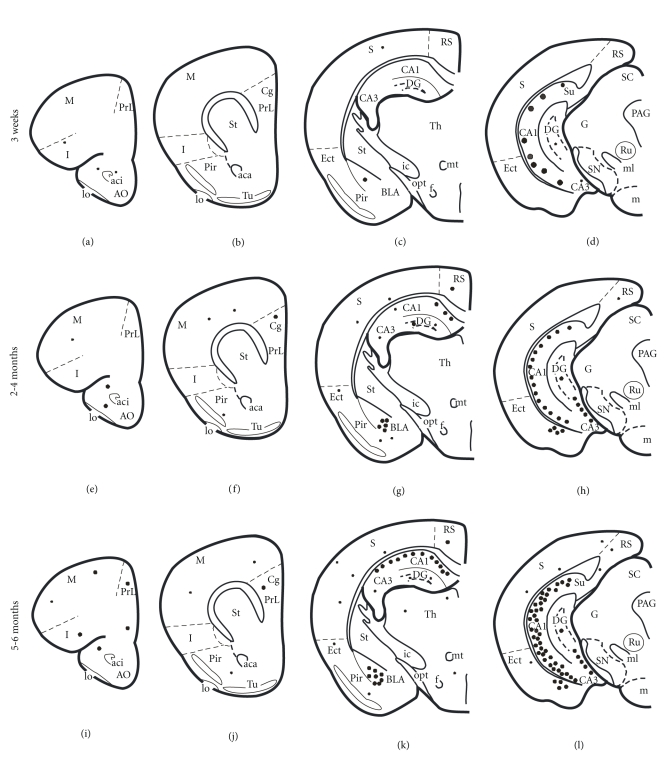
Schematic drawings showing the distribution of Alz50-ir neurons at 3-week, 2-, and 5-month-old transcardially-fixed 3xTg-AD mice. Alz50-ir neurons were found mainly in the CA1 ventral hippocampus at 3 weeks of age. Note the increase in the number of Alz50-ir hippocampal neurons in the 2- and 5-month old mutant mice. In addition there was an age-related increase in Alz-50 positive neurons within the BLA. Abbreviations are the same as in Figure 2. Small dots = 1–5 positive neurons, medium-size dots = 10–20 positive neurons, and large dots = 100 positive neurons.

**Figure 5:**
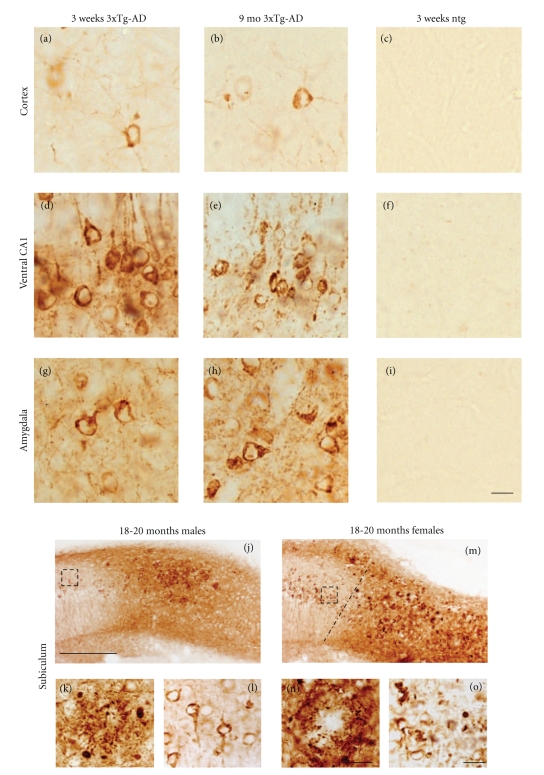
Photomicrographs of Alz50-ir neurons in 3-week, 9- and 18-20-month-old 3xTg-AD mice. At 3 weeks of age scattered neurons were found in the frontal parietal cortex (a) and BLA (g), whereas numerous Alz50-ir ventral hippocampal pyramidal cells were labeled (d). By 9 months of age, neurons in the BLA and ventral hippocampus appeared shrunken (e) compared to the cortex (b). Numerous Alz50-ir plaques and dystrophic neurites were found in the male ((j)-(k)) and female subiculum ((m)-(n)). Dashed boxes show higher magnification images of dystrophic Alz50-ir CA1 pyramidal neurons ((l), (o)). Dashed line in panel (m) demarks the CA1 field from the subiculum. Ntg mice failed to show Alz50 immunoreactivity ((c), (f), (i)). Scale bar in ((a)–(i)); ((k), (l), (o)) = 20 *μ*m, ((j), (m)) = 100 *μ*m, and (n) = 30 *μ*m.

The Alz50 antibody recognizes an early stage conformational tau epitope that labels neurons undergoing early degenerative events associated with NFT formation but has also been shown to label neurons in normal brains [33–37]. Similar to 6E10 staining, the number of Alz50-ir neurons increased with age in all regions examined (Figure 4). As early as 3 weeks of age Alz50-ir staining was seen predominantly within the more caudal and ventral aspects of the hippocampal CA1 pyramidal and subicular neurons, whereas a few scattered Alz50-ir neurons were found in the neocortex and BLA (Figures 4(a)–4(d)). In addition, Alz50-ir neurons were also found in the septal but not temporal portions of the hippocampal formation (data not shown). Beginning at 2–4 months of age a few scattered Alz50-ir neurons were found in layers 3 and 5 of the fronto-parietal and the deep layers of the cingulate and retrosplenial cortex in 3xTg-AD mice (Figures 4(e)–4(h)). By 5-6 months of age there was an apparent increase in the number of Alz50-ir neurons in the ventral hippocampus and amygdala (Figures 4(i)-4(l)), as well as more intense neuropil staining (Figures 1(q) and 1(r)). However, unlike the continuous distribution of 6E10-ir pyramidal neurons in the hippocampal complex, the topographic distribution of labeled Alz50-ir CA1 pyramidal neurons spread in dorsal and anterior directions from the ventral and caudal regions of the brain (Figure 4). At 3 weeks of age Alz50-ir neurons in the cortex, hippocampus, and amygdala appeared round or oval and displayed a dense rim of perinuclear staining (Figures 5(a), 5(d), and 5(g)) with long dendrites extending into the neuropil. At 9 months of age many Alz50-ir neurons appeared shrunken with a distorted cellular morphology in the hippocampal complex and amygdala (Figures 5(e) and 5(h)). Interestingly, some cells no longer displayed perinuclear staining but became completely filled with Alz50 reaction product (Figure 5(h)) or were dystrophic (data not shown). While the presence of Alz50-ir dystrophic neurites appeared concomitantly with the onset of A*β* plaque deposition between 8 and 9 months of age in female mutant mice, dramatic differences between male and female Alz50 staining were only apparent by 18–20 months of age. The dorsal and anterior subicular region adjacent to the CA1 neurons was most affected with Alz50-ir dystrophic neurites in male and female 18–20-month-old 3xTg-AD mice (Figures 5(j) and 5(m)). In general, numerous large and globular dystrophic Alz50-ir neurites were seen surrounded by plaques in both male and female mice (Figures 5(m) and 5(n)). In female transgenic mice many plaques displayed a rim of dark Alz50-ir dystrophic neurites with a clear center (Figure 5(k)), while in males this neuritic organization was less obvious (Figure 5(n)). In addition, in 18–20-month-old female 3xTg-AD mice dark, swollen, Alz50-ir dystrophic neurons were found in abundance among more lightly stained CA1 pyramidal cells (Figures 5(m) and 5(o)). Furthermore, the amygdala displayed similar neuronal perinuclear staining in male and female transgenic mice with an occasional rim of Alz50-ir dystrophic neurites surrounding plaques only in female mutant mice (data not shown).

#### 3.3.2. MC1 Immunoreactivity

**Figure 6:**
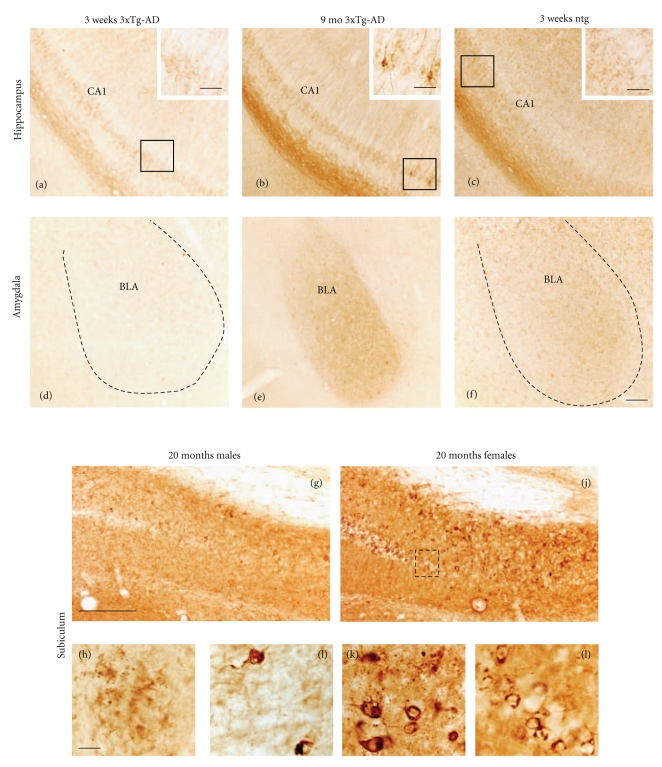
Photomicrographs showing MC1 immunoreactivity within the hippocampal CA1 field ((a)–(c)), BLA ((d)–(f)) and subiculum ((g)–(l)) at 3-week, 2–4-, 8-9-, and 20-month-old 3xTg-AD mice. Dashed line in (d) and (f) outlines the area containing the BLA. Note the increase in MC1 neuronal immunostaining in hippocampal CA1 neurons ((b), higher magnification shown in insert), the neuropil in the BLA (e) at 2–4 month and the reduction in reactivity in these areas in 9-month-old mutant mice ((c), higher magnification shown in insert and (f)). Low-magnification images of the subiculum from male (g) and female (j) 20 months of old 3xTg-AD mice. Example of MC1-ir neurites was observed in the subiculum of a male mutant mouse ((h)). Both male ((i)) and female ((j), higher magnification of boxed area shown in (l)) mutant mice displayed dystrophic subicular MC1-ir neurons. Scale bars: ((a)–(f)); ((g), (J)) =100 *μ*m, insets = 35 *μ*m, ((h), (i), (k), (l)) = 20 *μ*m.

The MC1 antibody recognizes early stage conformational epitopes similar to Alz50 but does not react with normal neuronal tau [34, 35, 37]. In contrast to Alz50 staining, lightly stained MC1-ir neurons were found only in the CA1 pyramidal cells of the ventral hippocampus in 3-week-old 3xTg-AD mice (Figure 6(a)). By 2–4 months, lightly stained MC1-ir neurons were seen throughout the CA1 subfield of the ventral hippocampus (Figure 6(b)). A few darkly stained MC1 neurons were seen, some of which appeared shrunken with blunted dendrites within the hippocampus. On the other hand, no MC1-ir neurons were seen in the BLA at 3 weeks of age (Figure 6(d)). In 2–4-month-old mutant mice, scattered round-or-oval shaped lightly stained MC1-ir neurons were found embedded within the MC1 immuopositive neuropil of the BLA. Interestingly, MC1-ir neuropil and neuronal labeling decreased in the hippocampus and BLA in 8-9-month-old mutant mice (Figures 6(c) and 6(f)). MC1-ir profiles were not detected in the neo- and limbic cortex up to 9 months of age. By 18–20 months, MC1 labeling matched Alz50 immunoreactivity in male and female 3xTg-AD mice. Although plaque-like structures were hard to detect, globular MC1-ir dystrophic neurites and neurons were found mainly throughout the female subiculum (Figures 6(j), 6(k), and 6(l)). Some dystrophic neurons appeared to have eccentrically located dark cytoplasmic neuronal inclusions (Figures 6(i), 6(k), and 6(l)).

#### 3.3.3. AT8 Immunoreactivity

**Figure 7:**
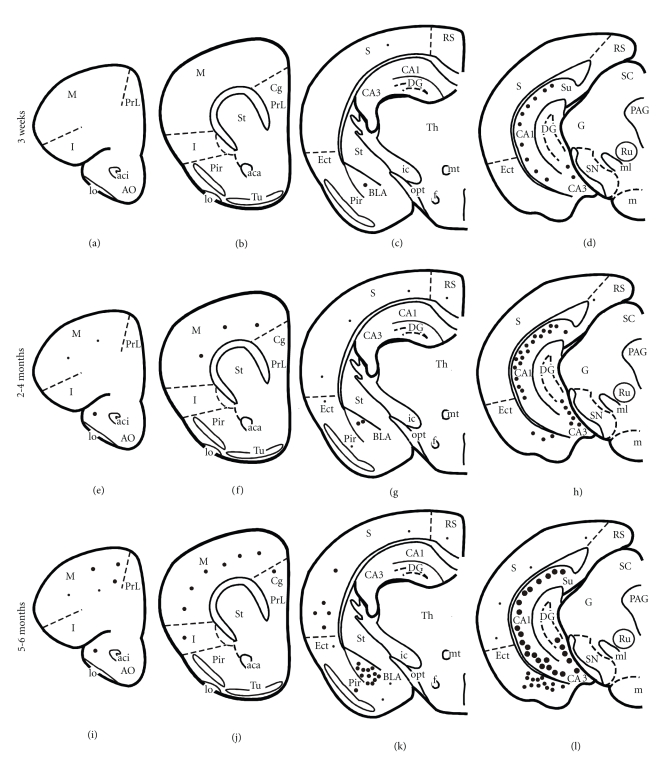
Schematic drawings showing the distribution of AT8-ir neurons in transcardially fixed 3-week, 3-, and 6-month-old 3xTg-AD mice. At 3 weeks, only scattered AT8-ir neurons were found in the cortex and basolateral amygdala compared to a greater number in the ventral hippocampus. Note that with increasing age, many more AT8-ir were found in the cortex, hippocampus, and amygdala. Abbreviations are the** s**ame as in Figure 2. Small dots = 1–5 positive neurons, medium-size dots = 10–20 positive neurons, and large dots = 100 positive neurons.

**Figure 8:**
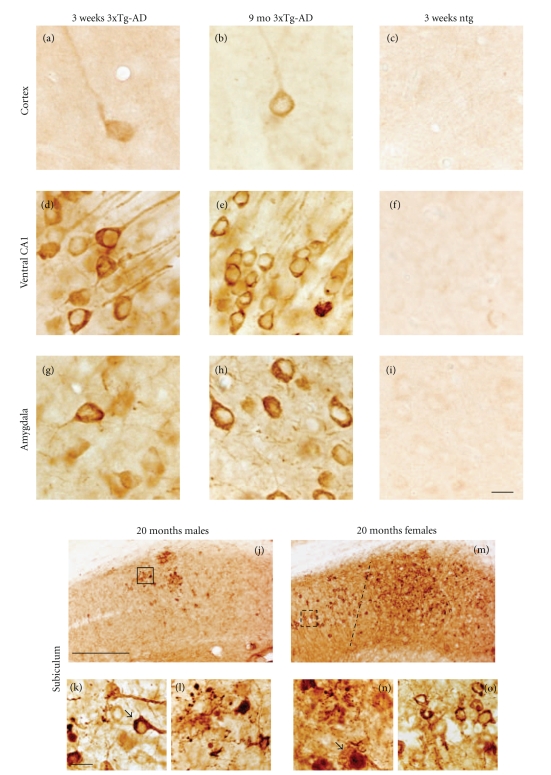
Photomicrographs showing AT8 immunoreactivity in the cortex ((a)–(c)), ventral CA1, ((d)–(f)), BLA ((g)–(i)), and subiculum ((j)–(o)) in 3-week, 9-, and 18–20-month-old 3xTg-AD mice. Transcardially fixed tissue revealed more AT8-ir neurons in the ventral hippocampus than either the neocortex or BLA in 3-week-old 3xTg-AD mice ((a), (d), (g)). Note that AT8-ir neurons within the hippocampus appeared shrunken with blunted dendrites at 9 months of age (e) when compared to younger (d) mutant mice. AT8 immunoreactive dystrophic neurons and plaques were observed in the subiculum of both male and female 20-month-old 3xTg-AD mice ((j)–(o)). Note the dystrophic neuronal morphology in male (k, arrow) and female (n, arrow) mice as well as the extent of tau pathology ((j), (m)). Dash line in m demarcates the boundary between the CA1 field and the subiculum. (l) and (o) represent higher magnification of boxed areas (j) and (m) respectively. Ntg 3-week-old mice displayed virtually no AT8 immunoreactivity ((c), (f), (i)). Scale bars: ((a)–(i)); ((k), (l), (n), (o)) = 20 *μ*m and (j), (m) = 100 *μ*m.

Hyperphosphorylated microtubule-associated protein tau is the major component of the paired helical filament seen in the NFT. The monoclonal antibody AT8 marks tau phosphorylation at both serine 202 and threonine 205 [29]. AT8-ir neurons were found in the neocortex, amygdala, and hippocampal formation in both male and female 3xTg-AD mice at all ages examined (Figure 7). Like the distribution of Alz50-ir neurons, hippocampal and subicular regions contained the highest number of AT8-ir neurons compared to the cortex and amygdala in 3-week-old 3xTg-AD mice (Figures 7(a)–7(d)). A few AT8-ir neurons were observed in layer 5 of the fronto-parietal cortex and BLA (Figures 7(a)–7(c)). As mutant mice age, the extent of neuronal AT8 labeling in the hippocampal complex increased in a ventral to dorsal and posterior to anterior gradient (Figures 7(e)–7(l)), but unlike the Alz50-ir profiles at 5-6 months of age, anterior portions of the hippocampal complex did not appear to be affected (Figures 7(c), 7(g), and 7(k)). At 3 weeks of age, AT8-ir neurons displayed more diffuse cytoplasmic staining in the cortex, hippocampus, and BLA (Figures 8(a), 8(d), and 8(g)), rather than the dense perinuclear staining observed in Alz50-ir neurons in these regions. However by 9 months of age AT8-ir neurons appeared shrunken, distorted, and showed thicker perinuclear staining, especially in the amygdala (Figures 8(b), 8(e), and 8(h)). Some neurons in the hippocampus (Figure 8(e)), BLA (Figure 8(h)), and subiculum displayed blunted AT8-ir dendrites. AT8 dystrophic neurons were more abundant in the female subiculum and constituted the greatest difference between male and female pathology at 18–20 months of age (Figures 8(j)–8(o)). While the entire extent of the female subiculum showed numerous AT8-ir dystrophic neurites, the male subiculum contained less neuritic dystrophy (Figure 8(j) versus Figure 8(m)). AT8-ir pyramidal cells displayed an asymmetric accumulation of dense perinuclear staining (Figures 8(k) and 8(o)), and AT8-ir dystrophic neurites were associated with plaques (Figures 8(l), 8(n)).

#### 3.3.4. AT180 Immunoreactivity

**Figure 9:**
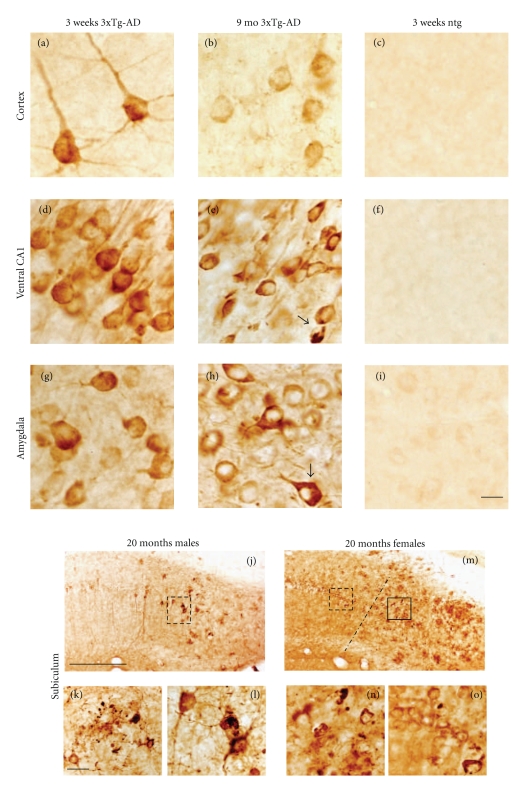
Photomicrographs showing AT180 immunoreactivity in the cortex ((a)–(c)), ventral CA1 ((d)–(F)), BLA ((g)–(i)), and subiculum ((j)–(o)) in 3xTg-AD mice. Note the more intense AT180-ir perinuclear staining (arrows), particularly in the BLA (h), and the shrunken appearance of ventral hippocampal neurons (e) at 9 months of age when compared to 3 weeks of age ((d), (g)). Greater AT180-ir tau pathology was observed in females than that in males at the CA1/subicular interface ((j), (m)). Higher magnification of dashed boxes in (j) and (m) are represented in (l) and (o), respectively, while (n) represents higher magnification of solid box in (m). Ntg mice showed only light background staining ((c), (f), (i)). Scale bars: ((a)–(i)); ((k), (l), (n), (o)) = 20 *μ*m and (j, m) = 100 *μ*m.

AT180 detects tau phosphorylation at the Thr231 site, an early event in the assembly of tau into filaments [27, 28]. AT180-ir neurons displayed the greatest number and spatial distribution of all tau antibodies examined at 3 weeks of age in 3xTg-AD mice. AT180-ir neurons were found in layer 5 of the fronto-parietal cortex, BLA, CA1 ventral hippocampus, and subiculum (Figures 9(a), 9(d), and 9(g)). Qualitatively there was an age-related increase in the number of AT180-ir neurons within each brain region examined as well as in the intensity of neuropil staining in the ventral hippocampus and BLA (Figures 1(s)–1(x)). At 3 weeks of age AT180 immunostaining appeared as diffuse cytoplasmic reactivity in round or oval pyramidal cells of the neocortex, hippocampus (Figure 9(a) and 9(d)) and BLA (Figure 9(g)) in 3xTg-AD mice. Some neurons embedded in the CA1 pyramidal cell layer, BLA, and subiculum displayed perinuclear staining (Figures 9(d) and 9(g)). By 9 months, AT180-ir hippocampal and BLA neurons revealed blunted staining in dendrites (Figure 9(e)). In the BLA, a number of neurons exhibited thickened perinuclear staining (Figure 9(h)). Differences between male and female AT180 staining occurred in the hippocampal CA1 neurons and the subiculum at older ages. Specifically, at 18–20 months of age AT180-ir dystrophic neurites were more widespread within the subiculum in female compared to male 3xTg-AD mice (Figures 9(j) and 9(m)). However, in male mice, AT180-ir dystrophic pathology was mainly found in the subiculum closest to the border with the CA1 pyramidal cell layer. In addition, CA1 pyramidal neurons displayed a dense perinuclear staining and blunted dendrites (Figures 9(l) and 9(o)).

#### 3.3.5. PHF-1 Immunoreactivity

**Figure 10:**
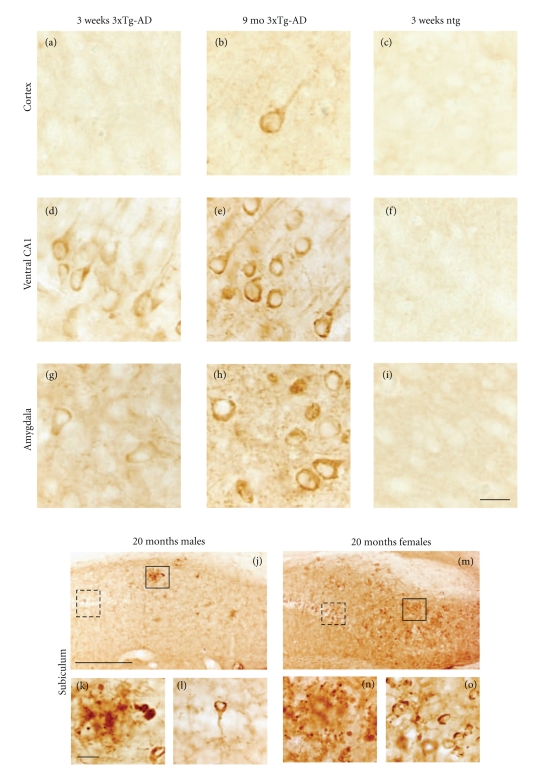
Photomicrographs showing PHF-1 immunostaining in cortex ((a)–(c)), hippocampus ((d)–(f)), BLA ((g)–(i)), and subiculum ((j)–(o)) in 3xTg-AD mice. PHF-1 immunostaining was seen almost exclusively in the ventral hippocampus of perfusion-fixed brains (d) compared to the cortex (a) and the BLA (g) at 3 weeks of age. By 8-9 months, many more PHF-1-ir neurons were apparent in the hippocampus and BLA ((e), (h)). Only scattered PHF-1-ir neurons were found in the neocortex at this age (b). Many more PHF-1 immunopositive neurons appeared in the dorsal anterior CA1 region of female mutant mice (area outline by dashed line in M and shown at higher magnification (o)) compared to males (area outline by dashed line in (j) and shown at higher magnification in (l)) at 18–20 months of age. PHF-1 immunopositive plaques were seen in the subiculum of male (solid boxed area in (j) and (k)) and female (solid boxed area in (m) and (o)) 3xTg-AD mice at 18–20 months of age. Note that the extent of PHF-1 immunoreactivity was greater in the subiculum in females (M) versus males (J). PHF-1-ir profiles were absent in ntg mice ((c), (f), (i)). Scale bars: (a)–(i); ((k), (l), (n), (o)) = 20 *μ*m and ((j), (m)) = 100 *μ*m.

PHF-1 recognizes tau phosphorylated at serine residues 396 and 404 and is generally considered a late marker in the evolution of PHF positive NFTs [30–32]. At 3 weeks of age only a few lightly stained PHF-1-ir neurons were observed in the CA1 region of the ventral hippocampus and the BLA in perfusion fixed 3xTg-AD mice (Figures 10(d) and 10(g)). At 2-3 months, a few PHF-ir neurons were seen at the CA1/subicular interface of the dorsal hippocampus (data not shown). By 9 months, the number of PHF-1-ir ventral hippocampal CA1 pyramidal and BLA neurons increased (Figures 10(e) and 10(h)). The distribution of PHF-ir neurons in the hippocampal complex followed a ventral to dorsal gradient. Up to 9 months of age, an occasional PHF-1-ir neuron was visible in the neocortex (Figure 10(b)). PHF-1 dystrophic neurons and neurites were more prevalent in the subiculum of aged females (Figures 10(m) and 10(n)) compared to aged males (Figures 10(j) and 10(k)). PHF-1-ir CA1 neurons displayed distorted cellular morphology, with broken, asymmetric, but strongly stained perinuclear staining in the female subiculum (Figure 10(o)). In contrast, PHF-1-ir neurons in the male subiculum showed similarly stained dark neurons but retained somatodendritic staining (Figure 10(l)).

### 3.4. Colocalization of 6E10 and Alz50 Immunoreactive Neurons

**Figure 11:**
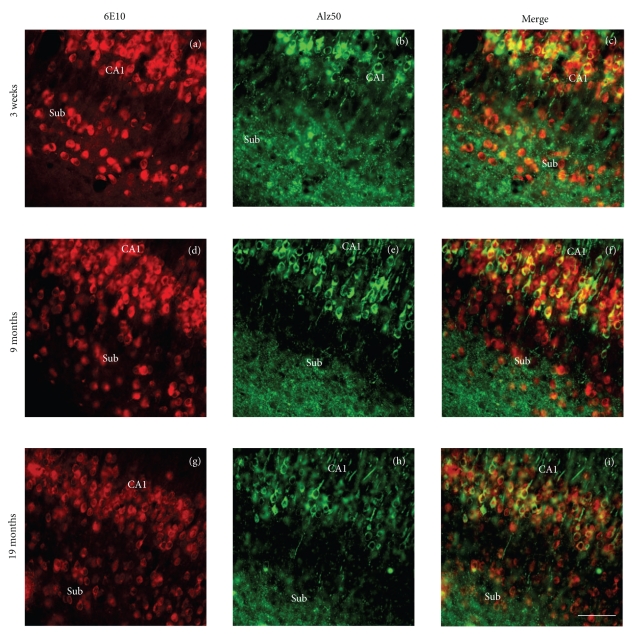
Immunofluorescent microscopic images showing single and dual 6E10 and Alz 50 staining in the hippocampal CA1 field and the subiculum of 3xTg-AD mice. Images of single 6E10 immunolabeled ((a), (d), and (g), red), (b), (e), (h) (Alz50, green) and merged (yellow, (c), (f), (i)) neurons at 3-week, 9- and 19-month-old 3xTg-AD mice. Note that 6E10 immunolabeled neurons are found in both the CA1 and subiculum but only Alz50 immunolabeled neurons were seen in CA1 at each age examined. The merged images show that many CA1 neurons contained both 6E10 and Alz50 immunoreactivity whereas the subicular neurons were only 6E10 positive at each age examined within Alz50-ir neuropil. Scale bar for (a)–(i) = 75 *μ*m.

To evaluate whether neurons expressing 6E10 also contained tau epitopes we performed dual immunofluorescence and comparative light microscopic experiments on adjacent hippocampal subicular sections at 3-week, 9-, and 18–20-month old male and female 3xTg-AD mice. Although it would have been interesting to double stain for 6E10 and other tau makers (AT180 and AT8), all three antibodies are murine IgG1s. Therefore, dual immunofluorescence experiments were performed only for 6E10 and Alz50, an IgM. In these experiments we observed two bands of 6E10 immnoreactivity located within ventrally located within CA1 hippocampal and subiculum neurons of female mutant mice at all ages examined (Figures 11(a), 11(d), and 11(g)). By contrast, Alz50-ir neurons were only observed in the CA1 pyramidal cell layer, and as neuropil staining in the subiculum (Figures 11(b), 11(e), and 11(h)). In general, Alz50-ir neurons were found to be colocalized with 6E10-ir neurons at each age examined in the ventral hippocampus (Figures 11(c), 11(f), and 11(i)).

**Figure 12:**
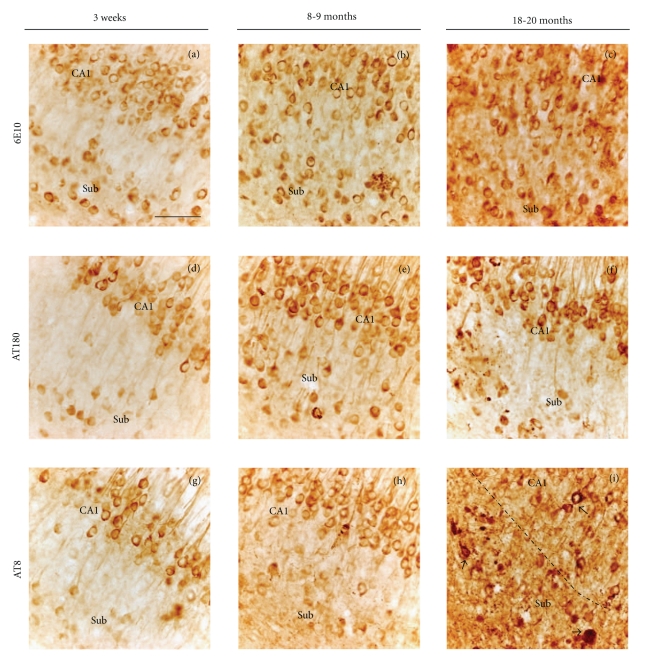
Light microscopic images of adjacent sections showing 6E10, AT180, and AT8 immunoreactive profiles in CA1 and subiculum in 3-week, 9-, and 18–20-months-old 3xTg-AD mice. AT180 and AT8 immunostained CA1 and subicular neurons were present at each age examined. This is in contrast to the Alz50, which only stained neurons in the CA1 field (see Figure 11). The arrows in F and I indicate dystrophic AT8 subicular neurons. Dashed line in (i) demarcates the CA1 field from the subiculum. Scale bar in (a) is the same for all panels = 50 *μ*m.

Because 6E10, AT180, or AT8 are all of the murine IgG1 subclass, adjacent sections through the CA1 and subicular fields were reacted with individual antibodies and visualized using peroxidase/DAB. In contrast to the single layer of Alz50-ir neurons in the CA1 pyramidal cell layer (Figures 12(b), 12(e), and 12(h)), numerous AT180, AT8, and 6E10 immunopositive neurons were seen in both CA1 and subicular fields in the ventral hippocampus at each age examined (Figure 12).

### 3.5. Ultrastructural Analysis of Amyloid and Tau Containing Structures in 3xTg-AD Mice

**Figure 13:**
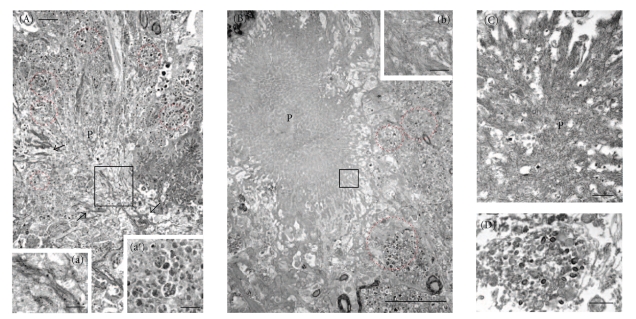
Electron microphotographs showing neuritic plaques (p) surrounded by numerous dystrophic neurites (dotted-red circles) in tissue sections through the hippocampal-subicular complex of a 9-(A) and 23-(B) month-old 3xTg-AD female mouse. Boxed area (a) shows details of the fibrillar material from amyloid plaque in A. a′ shows an example of a dystrophic neurite from a 9-month-old 3xTg-AD containing electron-dense multilaminar, multivesicular, and dense-core bodies similar to that seen in the cortex of a patient with AD (D). Note that at 23 months of age neuritic plaques show an electron dense center core (B) with numerous fibrillar branches (b) similar to a neuritic plaque seen in AD (c). Abbreviation: n: nucleus. Scale bars: A = 2 *μ*m, B = 5 *μ*m, C, a, b = 0.5 *μ*m, and D, a′ =1 *μ*m.

**Figure 14:**
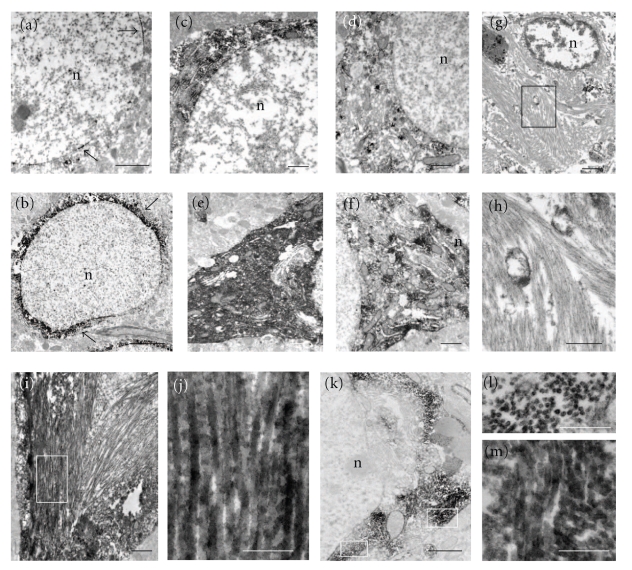
Electron photomicrographs showing perinuclear and cytoplasmic 6E10 immunoreactivity (black arrows) in hippocampal/subicular complex neurons at 2- (a) and 9- (b) month-old 3xTg-AD mice. Micrographs showing scattered Alz50 (c) and AT8 (d) immunoreactivity in hippocampal/subicular complex neurons in 2-month-old 3xTg-AD mice, whereas at 9 months Alz50 (e) and AT8 (f) immunoreactivity was increased, without the formation of filaments as seen in AD (g). Boxed area shows a higher-magnification photograph of the morphology of paired helical filaments in the AD brain (h). Photomicrographs of Alz50 ((i) and (j)) and AT8-ir ((k)–(m)) filaments in hippocampal/subicular complex neurons at 23 months in the 3xTg-AD mouse. J, Panel shows detail of straight Alz50-ir filaments from the white boxed area in (i). (l) and (m) areas containing AT8-ir filaments are outlined in white in panel k showing AT8 positive filaments in cross (l) and longitudinal (m) profile. Abbreviation: n: nucleus. Scale bars in (a), (b), (g) =2 *μ*m, (c), (d), (f), (k) = 1 *μ*m, (e), (i), (h) = 0.5 *μ*m and (j), (l), (m) = 200 nm.

At the ultrastructural level, unstained tissue sections revealed plaques within the hippocampal/subicular complex that displayed numerous fibrillar branches, which were surrounded by dystrophic neurites in 9-month-old 3xTg-AD mice (Figure 13(a)). In 23-month-old female 3xTg-AD mice, the majority of neuritic plaques displayed a central core surrounded by many more fibrillar branches and dystrophic neurites, similar to that seen in human AD plaques (Figure 13(c)). The neuropil contained dystrophic neurites of different sizes displaying various accumulations of electron-dense cytoplasmic material, multilaminar, multivesiclular, and dense-core bodies in both 9-and 23-month-old 3xTg-AD mice (Figures 13(a) and 13(b)), similar to that seen in an AD plaque (Figure 13(d)). TEM analysis of intraneuronal 6E10 immunoreactivity revealed staining mainly confined to the periphery of the nucleus within CA1 hippocampal/subicular neurons in 2-month-old 3xTg-AD mice (Figure 14(a)). At 9 months of age this perinuclear 6E10 reaction product appeared more intense and extended into the cytoplasm (Figure 14(b)).

At the ultrastructural level, Alz50 and AT8-ir were seen in the soma and dendrites of CA1 neurons in female 2-month-old mutant mice (Figures 14(c) and 14(d)). Intraneuronal electron dense Alz50 and AT8-ir cytoplasmic staining was more widespread and increased in intensity in 9 month-old-mutant mice (Figures 14(e) and 14(f)). Interestingly, cytoplasmic Alz50-ir appeared more extensive than AT8 immunoreactivity in these neurons at both 2 and 9 months of age (Figures 14(c), 14(d), 14(e), and 14(f)). Despite the presence of AT8 and Alz50-ir cytoplasmic labeling within hippocampal/subicular CA1 neurons, TEM failed to reveal any type of filamentous aggregates in 2- or 9-month-old 3xTg-AD mice (Figures 14(c), 14(d), 14(e), 14(f), 14(l), and 14(m)). In contrast, at 23 months of age both male and female 3xTg-AD mice displayed Alz50 and AT8-ir straight filaments with a 19–20 nm diameter within the hippocampal-subicular complex (Figures 14(i)–14(m)).

## 4. Discussion

Previous studies have shown widespread amyloid but not tau pathology at 6 months of age in the hippocampus [3, 12, 13, 17], and behavioral studies using the Morris Water Maze have demonstrated memory retention deficits dependent upon the appearance of amyloid pathology at 6- but not 2-month-old 3xTg-AD mice [11]. Our study found that (1) the appearance of 6E10 immunoreactivity was concomitant with conformation (Alz50 and MC1) and phosphorylation (AT8, AT180, and PHF-1) events in the neurons of the hippocampal-subicular complex and amygdala as early as 3 weeks of age; (2) the detection of the two phospho-epitopes AT8 and PHF-1 was fixation dependent at all ages; (3) the number of A*β* plaques, as well as neuritic and neuronal dystrophy, increased with age in the cortex, hippocampus, and subicular area in 3xTg-AD mice; (4) aged female mice displayed more plaque and tau pathology than aged male 3xTg-AD mice; a finally (5) straight tau filaments were found only in 23-month-old female 3xTg-AD mice. 

In the present study, 6E10-ir neurons were seen as early as 3 weeks of age in lamina 3 and 5 of the fronto-parietal cortex, deep layers of the cingulate cortex, CA1 pyramidal neurons of the hippocampus, subiculum, and BLA in 3xTg-AD mice independent of gender. These data indicate that intraneuronal 6E10 immunoreactivity occurs extremely early in the development of these mice (3 weeks) and are in contrast to previous investigations showing that 6E10 intraneuronal staining occurred between 3 and 6 months of age in the hippocampus of these mice [12, 17]. Because the 6E10 epitope resides in amino acids 3–8 of the N-terminal portion of the A*β* sequence, it does not preclude binding to full length APP or its amyloidgenic derivatives. Consequently, the exact amyloid species revealed within neurons at 3 weeks of age in 3xTg-AD mice and at other ages examined is unclear. Oligomeric A*β* species may, in part, underlie cellular degeneration [3, 11, 38–48].

We also observed many shrunken 6E10-ir neurons beginning at 8-9 month of age in each brain area examined in both male and female 3xTg-AD mice. This phenotype was most pronounced in CA1 hippocampal neurons of older female 3xTg-AD mice. These neurons exhibited loss of dendritic 6E10 immunoreactivity when compared to CA1 neurons in male mutant mice. These gender differences in A*β* intraneuronal pathology lend support to prior investigations showing that female 3xTg-AD mice display earlier and more severe plaque pathology [16], which may be related to progesterone and estrogen-mediated signaling [49]. However, other factors may contribute to the discrepancies in AD-like pathology reported in studies using different colonies of 3xTg-AD mice, including a loss of phenotype related to successive breeding, founder effects between colonies, or differential expression of transgenes [16]. Together or separately, variations in transgenic animal strain or amyloid species may affect both pathological and behavioral observations reported in studies using 3xTg-AD mice.

Of interest was the observation that the immunohistochemical localization of phosphospecific tau proteins AT8 and PHF-1 was fixation dependent, whereas Alz50, MC1, AT180, and 6E10 immunoreactivity was fixation independent in young, juvenile, and middle age 3xTg-AD mice. Notably, neurons containing the phospho-epitope AT8 were seen in layer 5 of frontoparietal cortex, ventral hippocampal pyramidal, and amygdala neurons, whereas PHF-1-ir intraneuronal staining was seen only in the ventral hippocampus in perfusion-fixed 3-week-old 3xTg-AD mice. Previous biochemical and immunohistochemical studies indicate that fixative composition affects the ability to detect various cytoskeletal proteins [18, 22, 23]. The process of aldehyde fixation relies upon a slow cross-linking of carbonyl aldehyde to functional protein groups. While perfusion fixation rapidly exposes proteins to fixative through the vasculature, immersion fixation relies upon a slow 1 mm per hour diffusion rate to penetrate tissue [20]. It is possible during immersion fixation that proteolysis or dephosphorylation of the tau phospho-epitopes recognized by AT8 and PHF-1 occurs prior to the full penetration of the fixative. Since immersion-fixed tissue reacts with C-terminal, N-terminal, and internal tau antibodies, proteoloysis of tau is unlikely [25, 50]. A more plausible possibility is the destruction of AT8 and PHF-1 epitopes by the action of endogenous phosphatases during slow immersion fixation [51–53]. However, AT180 immunoreactivity remained robust even in immersion-fixed tissue. Likely, phosphatases act differentially depending on the individual tau phosphoepitopes [54]; hence, it is possible that the AT180 phospho-epitope is less sensitive to phosphatase activity than either AT8 or PHF-1 phosphorylation sites, resulting in a more robust staining in immersion-fixed tissue. Although long postmortem intervals reduce phospho-tau antigenicity due to active phosphatases, [19, 50, 55], this was not a confounding variable in the present study since all animals were sacrificed and perfused with fixative within 5 minutes of anesthetization. The potential effect of fixation on the immunolocalization of tau and other proteins [56] represents an important caveat that may provide an explanation for the underlying differences in tau antigenicity reported in previous studies using the 3xTg-AD mouse model [3, 12–14, 16, 57, 58]. 

Although a previous report described intraneuronal MC1-ir CA1 dorsal hippocampal neurons only in 12–15-month-old 3xTg-AD mice [12], the present study displayed weak MC1 immunoreactive neurons in the ventral hippocampus even as early as 3 weeks of age. We also observed Alz50 immunoreactive CA1 pyramidal and subicular neurons in the ventral aspect of the hippocampal/subicular complex at 3 weeks of age. Although both MC1 and Alz50 antibodies recognize similar epitopes, they are not identical. Overall, Alz50 immunoreactivity was much more intense and labeled a greater distribution of immunoreactive neurons than with the MC1 antibody. While Alz50 immunoreactivity increased in an age dependent manner in the neocortex, hippocampus, and amygdala, MC1 immunoreactivity exhibited a biphasic response, peaking initially between 2 and 4 months, declining at 9 months and then peaking again at 18–20 months in numerous swollen, tangle-like appearing CA1 hippocampal and subicular neurons. Interestingly, both MC1 and Alz50-ir neurons were found in the CA1 pyramidal but not the subicular cell layer in the ventral hippocampal formation at the ages examined suggesting that Alz50 is labeling similar populations of neurons.

Unlike previous studies where various phosphotau epitopes were immunohistochemically detectable by 15 months of age in 3xTg-AD mice [12, 17, 49], we observed tau-ir neurons primarily in the ventral CA1 hippocampal pyramidal and subicular neurons at 3-weeks of age. This occurred well before the onset of plaque deposition in 8-9-month-old 3xTg-AD mice [17]. The current findings indicate that intraneuronal amyloid and tau co-occur or appear concurrent in the same neurons at least as early as 3 weeks of age suggesting that these mutant mice are born with the dual expression of these proteins within select populations of brain cells. The early appearance of tau-ir neurons in the forebrain of 3xTg-AD mice is not surprising since overexpression of mutant P301L four repeat tau and the APP_swe_ amyloid mutation together [59] or tau P301L alone [60] induces an age dependent onset of MC1, AT8, and PHF-ir neurofibrillary tangles (NFTs) in the telencephalon of mutant mice as young as 2.5 months of age [60].

However, it remains to be determined whether amyloid interacts with tau over time to induce the apparent age-related perikaryal degeneration seen in these mice. Studies are underway to test the hypothesis that depletion of intraneuronal amyloid at an early age would prevent or slow the degeneration of neurons in these mice. It is interesting to note that tau containing straight filaments were seen in 18–20-month-old mutant mice suggesting that late-stage tau neuronal aggregation also plays a key role in the ultimate demise of neurons in 3xTg-AD mice. Although no studies to date have shown frank neuronal cell loss, a recent report described a reduction in the number of neurons containing 6E10 and tau markers in the brainstem of these mutant mice [15].

To more fully understand the vulnerability of hippocampal complex neurons, we colocalized 6E10 and Alz50 using immunofluorescence. A previous study colocalized HT7, a human specific pan tau antibody and M71/3 (oligomeric A*β*) in the dorsal hippocampus in 3xTg-AD mice [3]. However, at earlier ages conformation or phosphospecific tau antibodies were not evaluated. Our immunofluorescence colocalization experiments demonstrated that virtually all ventral CA1 pyramidal but not all subicular neurons contained both Alz50 and 6E10 from 3 weeks to at least 18–20 months of age, suggesting that CA1 neurons are more vulnerable to Alz50 pathology. Although we were unable to perform colocalization experiments between 6E10 and our tau antibodies, we performed a comparative light microscopic analysis of adjacent hippocampal sections. This study revealed AT180 and AT8-ir neurons in the caudal aspects of the subicular region where we failed to visualize Alz50-ir neurons across all ages examined. The reason for the selective appearance of AT180 and AT8 in the ventral subiculum remains unknown. One possibility is that neurofibrillary pathology proceeds according to a sequence of tau conformational and phosphorylation events [61–64], which may vary between neuronal populations. In AD, as tau transitions to a more hyperphosphorylated state, it undergoes a self-assembly process forming PHF structures reducing the microtubule stability of the neuronal cytoskeleton [65, 66]. More specifically, the evolution of NFTs in AD follows a progression of phosphorylated tau modifications from TG3 (phosphospecific at Thr231), found in preneurofibrillary tangles (preNFTs), to AT8 and PHF-1 in intraneuronal and extraneuronal NFTs [61]. This progression may be dynamic since intraneuronal AT180 and AT8 precedes Alz50 tau formation before the appearance of fibrillar tau [63, 64]. We suggest that the sequence of cellular tau conformation and phosphorylation events may also be dynamic in 3xTg-AD mice since Alz50-ir neurons were found only in CA1 neurons in the hippocampus but not in the underlying subiculum.

Despite differences in tau intraneuronal epitope expression, we found age-related changes in neuronal morphology in the 3xTg-AD mouse to be similar to the NFT neuronal phenotypes described in the human AD brain [67, 68]. Some AT8-ir neurons at 9 months of age displayed blunted dendrites (see Figure 8(e)) or tortuous proximal dendrites and thickened perinuclear staining reminiscent of Braak type 2 neurons [67]. In our aged mutant mice, proximal and distal dendrites lost AT8 immunoreactivity and neurons appeared more globose and dystrophic similar to Braak type 3 neurons, which accumulate fibrillar material in the human AD brain [67]. Furthermore, the present ultrastructural analysis revealed, for the first time, the presence of AT8 and Alz50 positive straight tau filamentous aggregates in female aged 23-month-old, but not in young or middle-age 3xTg-AD mice of the same gender, which are similar to those seen in tangle bearing neurons in the human AD brain. Similar tau filaments have been described in single transgenic mice overexpressing the mutant P301L human tau gene [10, 59] as well as in the double mutant (TAPP) mouse expressing APP_swe_ and P301L mutant forms of tau [59]. NFT formation was enhanced in young and middle age TAPP mutant mice with respect to single tau transgenic mice [59]. Although young and middle-aged 3xTg-AD mice carry the same APP_swe_, and Tau_P301L_ mutations as the TAPP mouse, 3xTg-AD mice did not accumulate filamentous aggregates at younger ages. In this regard, mice overexpressing human genomic and cDNA tau genes also do not display intraneuronal filamentous structures at young ages [69]. These findings suggest that factors other than the accumulation of tau underlie the filamentous tangle bearing phenotype seen in human AD. Factors that may influence the formation of AD filaments include differences in the abundance or ratio of specific tau isotypes [70] in different cohorts of neurons and/or minor alterations in proteases, kinases, phosphatases that alter the structure of these proteins during the life span of the neuron [25], and/or the promoter system designed for specific transgene expression in particular brain regions. Since age is a major risk factor for AD, aging itself may be a crucial variable in the formation of intraneuronal filaments in 3xTg-AD mice. Despite differences in the evolution of tau biochemistry, there are morphological similarities between neurons containing tau in 3xTg-AD mice and human AD indicating the usefulness of these mice for studies of the mechanism(s) underlying select aspects of AD pathology.

## Figures and Tables

**Table 1:** Summary of Antibodies and Characterisitcs.

Primary Antibody	Source	Epitope and Isotype	Secondary Antibody	Blocking Serum
Alz50	Dr. Peter Davies Albert Einstein College of Medicine, NY	aa's 5–15, 312–322;IgM	Goat-Anti mouse IgG, Vector Labs, Burlingame, CA	Goat; Gemini Bio-Products, West Sacramento, CA
MC1	Dr. Peter Davies Albert Einstein College of Medicine, NY	aa's 5–15, 312–322;IgG1	Goat-Anti mouse IgG, Vector Labs, Burlingame, CA	Goat; Gemini Bio-Products, West Sacramento, CA
AT180	ThermoFisher, Walthman, MA	Phosphothreonine 231; IgG 1K	Goat-Anti mouse IgG, Vector Labs, Burlingame, CA	Goat; Gemini Bio-Products, West Sacramento, CA
AT8	ThermoFisher, Walthman, MA	Phosphoserine 202/205*; *IgG 1	Goat-Anti mouse IgG, Vector Labs, Burlingame, CA	Goat; Gemini Bio-Products, West Sacramento, CA
PHF-1	Dr. Peter Davies Albert Einstein College of Medicine, NY	Phosphoserine 396/404*; *IgG1	Goat-Anti mouse IgG, Vector Labs, Burlingame, CA	Goat; Gemini Bio-Products, West Sacramento, CA
A*β*/APP (6E10)	Covance Princeton,NJ	aa's 3–8 of A*β* seqeunce; IgG 1		

## References

[1] Eriksen JL, Janus CG (2007). Plaques, tangles, and memory loss in mouse models of neurodegeneration. *Behavior Genetics*.

[2] Games D, Adams D, Alessandrini R (1995). Alzheimer-type neuropathology in transgenic mice overexpressing V717F *β*-amyloid precursor protein. *Nature*.

[3] Oddo S, Caccamo A, Tran L (2006). Temporal profile of amyloid-*β* (A*β*) oligomerization in an in vivo model of Alzheimer disease: a link between A*β* and tau pathology. *Journal of Biological Chemistry*.

[4] Götz J, Deters N, Doldissen A (2007). A decade of tau transgenic animal models and beyond. *Brain Pathology*.

[5] Götz J, Streffer JR, David D (2004). Transgenic animal models of Alzheimer’s disease and related disorders: histopathology, behavior and therapy. *Molecular Psychiatry*.

[6] Spires TL, Hyman BT (2005). Transgenic models of Alzheimer’s disease: learning from animals. *NeuroRx*.

[7] Allen B, Ingram E, Takao M (2002). Abundant tau filaments and nonapoptotic neurodegeneration in transgenic mice expressing human P301s tau protein. *Journal of Neuroscience*.

[8] Götz J, Chen F, Barmettler R, Nitsch RM (2001). Tau filament formation in transgenic mice expressing P301L tau. *Journal of Biological Chemistry*.

[9] Lewis J, McGowan E, Rockwood J (2000). Neurofibrillary tangles, amyotrophy and progressive motor disturbance in mice expressing mutant (P301L)tau protein. *Nature Genetics*.

[10] Lin W-L, Lewis J, Yen S-H, Hutton M, Dickson DW (2003). Ultrastructural neuronal pathology in transgenic mice expressing mutant (P301L) human tau. *Journal of Neurocytology*.

[11] Billings LM, Oddo S, Green KN, McGaugh JL, LaFerla FM (2005). Intraneuronal A*β* causes the onset of early Alzheimer’s disease-related cognitive deficits in transgenic mice. *Neuron*.

[12] Oddo S, Caccamo A, Kitazawa M, Tseng BP, LaFerla FM (2003). Amyloid deposition precedes tangle formation in a triple transgenic model of Alzheimer’s disease. *Neurobiology of Aging*.

[13] Oddo S, Caccamo A, Shepherd JD (2003). Triple-transgenic model of Alzheimer’s disease with plaques and tangles: intracellular A*β* and synaptic dysfunction. *Neuron*.

[14] Oddo S, Caccamo A, Smith IF, Green KN, LaFerla FM (2006). A dynamic relationship between intracellular and extracellular pools of A*β*. *American Journal of Pathology*.

[15] Overk CR, Kelley CM, Mufson EJ (2009). Brainstem Alzheimer’s-like pathology in the triple transgenic mouse model of Alzheimer’s disease. *Neurobiology of Disease*.

[16] Hirata-Fukae C, Li H-F, Hoe H-S (2008). Females exhibit more extensive amyloid, but not tau, pathology in an Alzheimer transgenic model. *Brain Research*.

[17] Mastrangelo MA, Bowers WJ (2008). Detailed immunohistochemical characterization of temporal and spatial progression of Alzheimer’s disease-related pathologies in male triple-transgenic mice. *BMC Neuroscience*.

[18] Conti CJ, Larcher F, Chesner J, Aldaz CM (1988). Polyacrylamide gel electrophoresis and immunoblotting of proteins extracted from paraffin-embedded tissue sections. *Journal of Histochemistry and Cytochemistry*.

[19] Matsuo ES, Shin R-W, Billingsley ML (1994). Biopsy-derived adult human brain tau is phosphorylated at many of the same sites as Alzheimer’s disease paired helical filament tau. *Neuron*.

[20] Fox CH, Johnson FB, Whiting J, Roller PP (1985). Formaldehyde fixation. *Journal of Histochemistry and Cytochemistry*.

[21] Pollock NJ, Wood JG (1988). Differential sensitivity of the microtubule-associated protein, tau, in Alzheimer’s disease tissue to formalin fixation. *Journal of Histochemistry and Cytochemistry*.

[22] Riederer BM (1989). Antigen preservation tests for immunocytochemical detection of cytoskeletal proteins: influence of aldehyde fixatives. *Journal of Histochemistry and Cytochemistry*.

[23] Riederer BM, Porchet R, Marugg RA, Binder LI (1993). Solubility of cytoskeletal proteins in immunohistochemistry and the influence of fixation. *Journal of Histochemistry and Cytochemistry*.

[24] Trojanowski JQ, Obrocka MA, Lee VM-Y (1985). Distribution of neurofilament subunits in neurons and neuronal processes: immunohistochemical studies of bovine cerebellum with subunit-specific monoclonal antibodies. *Journal of Histochemistry and Cytochemistry*.

[25] Trojanowski JQ, Schuck T, Schmidt ML, Lee VM-Y (1989). Distribution of tau proteins in the normal human central and peripheral nervous system. *Journal of Histochemistry and Cytochemistry*.

[26] Vickers JC, Riederer BM, Marugg RA (1994). Alterations in neurofilament protein immunoreactivity in human hippocampal neurons related to normal aging and Alzheimer’s disease. *Neuroscience*.

[27] Goedert M, Jakes R, Crowther RA (1994). Epitope mapping of monoclonal antibodies to the paired helical filaments of Alzheimer’s disease: identification of phosphorylation sites in tau protein. *Biochemical Journal*.

[28] Mercken M, Vandermeeren M, Lubke U (1992). Monoclonal antibodies with selective specificity for Alzheimer Tau are directed against phosphatase-sensitive epitopes. *Acta Neuropathologica*.

[29] Goedert M, Jakes R, Vanmechelen E (1995). Monoclonal antibody AT8 recognises tau protein phosphorylated at both serine 202 and threonine 205. *Neuroscience Letters*.

[30] Greenberg SG, Davies P (1990). A preparation of Alzheimer paired helical filaments that displays distinct *τ* proteins by polyacrylamide gel electrophoresis. *Proceedings of the National Academy of Sciences of the United States of America*.

[31] Lang E, Szendrei GI, Lee VM-Y, Otvos L (1992). Immunological and conformational characterization of a phosphorylated immunodominant epitope on the paired helical filaments found in Alzheimer’s disease. *Biochemical and Biophysical Research Communications*.

[32] Otvos L, Feiner L, Lang E, Szendrei GI, Goedert M, Lee VM-Y (1994). Monoclonal antibody PHF-1 recognizes tau protein phosphorylated at serine residues 396 and 404. *Journal of Neuroscience Research*.

[33] Carmel G, Mager EM, Binder LI, Kuret J (1996). The structural basis of monoclonal antibody Alz50’s selectivity for Alzheimer’s disease pathology. *Journal of Biological Chemistry*.

[34] Jicha GA, Berenfeld B, Davies P (1999). Sequence requirements for formation of conformational variants of tau similar to those found in Alzheimer’s disease. *Journal of Neuroscience Research*.

[35] Jicha GA, Bowser R, Kazam IG, Davies P (1997). Alz-50 and MC-1, a new monoclonal antibody raised to paired helical filaments, recognize conformational epitopes on recombinant tau. *Journal of Neuroscience Research*.

[36] Wolozin BL, Pruchnicki A, Dickson DW, Davies P (1986). A neuronal antigen in the brains of Alzheimer patients. *Science*.

[37] Rye DB, Leverenz J, Greenberg SG, Davies P, Saper CB (1993). The distribution of Alz-50 immunoreactivity in the normal human brain. *Neuroscience*.

[38] Yan SD, Yan SF, Chen X (1995). Non-enzymatically glycated tau in Alzheimer’s disease induces neuronal oxidant stress resulting in cytokine gene expression and release of amyloid *β*-peptide. *Nature Medicine*.

[39] De Felice FG, Velasco PT, Lambert MP (2007). A*β* oligomers induce neuronal oxidative stress through an N-methyl-D-aspartate receptor-dependent mechanism that is blocked by the Alzheimer drug memantine. *Journal of Biological Chemistry*.

[40] De Felice FG, Wu D, Lambert MP (2008). Alzheimer’s disease-type neuronal tau hyperphosphorylation induced by A*β* oligomers. *Neurobiology of Aging*.

[41] Ferreira ST, Vieira MNN, De Felice FG (2007). Soluble protein oligomers as emerging toxins in Alzheimer’s and other amyloid diseases. *IUBMB Life*.

[42] Glabe CG (2008). Structural classification of toxic amyloid oligomers. *Journal of Biological Chemistry*.

[43] Lacor PN, Buniel MC, Furlow PW (2007). A*β* oligomer-induced aberrations in synapse composition, shape, and density provide a molecular basis for loss of connectivity in Alzheimer’s disease. *Journal of Neuroscience*.

[44] Lambert MP, Velasco PT, Chang L (2007). Monoclonal antibodies that target pathological assemblies of A*β*. *Journal of Neurochemistry*.

[45] Shankar GM, Bloodgood BL, Townsend M, Walsh DM, Selkoe DJ, Sabatini BL (2007). Natural oligomers of the Alzheimer amyloid-*β* protein induce reversible synapse loss by modulating an NMDA-type glutamate receptor-dependent signaling pathway. *Journal of Neuroscience*.

[46] Walsh DM, Hartley DM, Condron MM, Selkoe DJ, Teplow DB (2001). In vitro studies of amyloid *β*-protein fibril assembly and toxicity provide clues to the aetiology of Flemish variant (Ala692 → Gly) Alzheimer’s disease. *Biochemical Journal*.

[47] Walsh DM, Selkoe DJ (2007). A*β* oligomers—a decade of discovery. *Journal of Neurochemistry*.

[48] Hirata-Fukae C, Li H-F, Ma L (2009). Levels of soluble and insoluble tau reflect overall status of tau phosphorylation in vivo. *Neuroscience Letters*.

[49] Carroll JC, Rosario ER, Chang L (2007). Progesterone and estrogen regulate Alzheimer-like neuropathology in female 3xTg-AD mice. *Journal of Neuroscience*.

[50] Garver TD, Harris KA, Lehman RAW, Lee VM-Y, Trojanowski JQ, Billingsley ML (1994). *τ* Phosphorylation in human, primate, and rat brain: evidence that a pool of *τ* is highly phosphorylated in vivo and is rapidly dephosphorylated in vitro. *Journal of Neurochemistry*.

[51] Trojanowski JQ, Lee VM-Y (1995). Phosphorylation of paired helical filament tau in Alzheimer’s disease neurofibrillary lesions: focusing on phosphatases. *FASEB Journal*.

[52] Liang Z, Liu F, Iqbal K, Grundke-Iqbal I, Wegiel J, Gong C-X (2008). Decrease of protein phosphatase 2A and its association with accumulation and hyperphosphorylation of tau in Down syndrome. *Journal of Alzheimer’s Disease*.

[53] Wang J-Z, Grundke-Iqbal I, Iqbal K (2007). Kinases and phosphatases and tau sites involved in Alzheimer neurofibrillary degeneration. *European Journal of Neuroscience*.

[54] Liu F, Grundke-Iqbal I, Iqbal K, Gong C-X (2005). Contributions of protein phosphatases PP1, PP2A, PP2B and PP5 to the regulation of tau phosphorylation. *European Journal of Neuroscience*.

[55] Soulié C, Lépagnol J, Delacourte A, Caillet-Boudin ML (1996). Dephosphorylation studies of SKNSH-SY 5Y cell Tau proteins by endogenous phosphatase activity. *Neuroscience Letters*.

[56] Lavenex P, Lavenex PB, Bennett JL, Amaral DG (2009). Postmortem changes in the neuroanatomical characteristics of the primate brain: hippocampal formation. *Journal of Comparative Neurology*.

[57] Movsesyan N, Ghochikyan A, Mkrtichyan M (2008). Reducing AD-like pathology in 3xTg-AD mouse model by DNA epitope vaccine—a novel immunotherapeutic strategy. *PLoS ONE*.

[58] Oddo S, Billings L, Kesslak JP, Cribbs DH, LaFerla FM (2004). A*β* immunotherapy leads to clearance of early, but not late, hyperphosphorylated tau aggregates via the proteasome. *Neuron*.

[59] Lewis J, Dickson DW, Lin W-L (2001). Enhanced neurofibrillary degeneration in transgenic mice expressing mutant tau and APP. *Science*.

[60] Ramsden M, Kotilinek L, Forster C (2005). Age-dependent neurofibrillary tangle formation, neuron loss, and memory impairment in a mouse model of human tauopathy (P301L). *Journal of Neuroscience*.

[61] Augustinack JC, Schneider A, Mandelkow E-M, Hyman BT (2002). Specific tau phosphorylation sites correlate with severity of neuronal cytopathology in Alzheimer’s disease. *Acta Neuropathologica*.

[62] Binder LI, Guillozet-Bongaarts AL, Garcia-Sierra F, Berry RW (2005). Tau, tangles, and Alzheimer’s disease. *Biochimica et Biophysica Acta*.

[63] Luna-Muñoz J, Chávez-Macías L, García-Sierra F, Mena R (2007). Earliest stages of tau conformational changes are related to the appearance of a sequence of specific phospho-dependent tau epitopes in Alzheimer’s disease. *Journal of Alzheimer’s Disease*.

[64] Luna-Muñoz J, García-Sierra F, Falcón V, Menéndez I, Chávez-Macías L, Mena R (2005). Regional conformational change involving phosphorylation of tau protein at the Thr231, precedes the structural change detected by Alz-50 antibody in Alzheimer’s disease. *Journal of Alzheimer’s Disease*.

[65] Alonso ADC, Zaidi T, Novak M, Grundke-Iqbal I, Iqbal K (2001). Hyperphosphorylation induces self-assembly of *τ* into tangles of paired helical filaments/straight filaments. *Proceedings of the National Academy of Sciences of the United States of America*.

[66] Goedert M (2005). Tau gene mutations and their effects. *Movement Disorders*.

[67] Braak E, Braak H, Mandelkow E-M (1994). A sequence of cytoskeleton changes related to the formation of neurofibrillary tangles and neuropil threads. *Acta Neuropathologica*.

[68] Sassin I, Schultz C, Thal DR (2000). Evolution of Alzheimer’s disease-related cytoskeletal changes in the basal nucleus of Meynert. *Acta Neuropathologica*.

[69] Duff K, Knight H, Refolo LM (2000). Characterization of pathology in transgenic mice over-expressing human genomic and cDNA tau transgenes. *Neurobiology of Disease*.

[70] Ginsberg SD, Che S, Counts SE, Mufson EJ (2006). Shift in the ratio of three-repeat tau and four-repeat tau mRNAs in individual cholinergic basal forebrain neurons in mild cognitive impairment and Alzheimer’s disease. *Journal of Neurochemistry*.

